# Investigation of somatic *NKX2-5* mutations in congenital heart disease

**DOI:** 10.1136/jmg.2008.060277

**Published:** 2009-01-23

**Authors:** J M Draus, M A Hauck, M Goetsch, E H Austin, A Tomita-Mitchell, M E Mitchell

**Affiliations:** 1 Department of Surgery, University of Louisville School of Medicine, Louisville, Kentucky, USA; 2 Department of Surgery, Division of Cardiothoracic Surgery, University of Louisville School of Medicine, Louisville, Kentucky, USA; 3 Department of Surgery, Division of Pediatric Cardiothoracic Surgery, Medical College of Wisconsin, Milwaukee, Wisconsin, USA

## Abstract

**Background:**

Reports of somatic mutations found in hearts with cardiac septal defects have suggested that these mutations are aetiologic in pathologic cardiac development. However, the hearts in these reports had been fixed in formalin for over 22 years. Because of the profound implication of this finding, we attempted to replicate it using fresh frozen tissue obtained in the current era from 28 patients with septal defects who underwent cardiac surgery and who were enrolled in our congenital heart disease tissue bank.

**Methods:**

Our cohort included patients with atrial septal defects (ASD, n = 13), ventricular septal defects (VSD, n = 5), and atrioventricular canal defects (AVCD, n = 10). Cardiac tissue samples were collected both from diseased tissue located immediately adjacent to the defect and from anatomically normal tissue located at a site remote from the defect (right atrial appendage). Tissue samples were immediately frozen in liquid nitrogen and stored at −80°C. Genomic DNA was isolated and amplified using the same methodology described in the previously published reports. 42 pathologic cardiac tissue samples were sequenced.

**Results:**

One non-synonymous germline sequence variant was identified in one patient. Two synonymous germline sequence variants were identified in two separate patients. A common single nucleotide polymorphism (SNP) was identified in 16 patients. Based on the incidence of somatic mutations described in the previously published reports, our study was adequately powered to replicate the previous studies. No evidence of somatic mutations was found in this study.

**Conclusion:**

Somatic mutations in *NKX2-5* do not represent an important aetiologic pathway in pathologic cardiac development in patients with cardiac septal defects.

Congenital heart disease (CHD) is a common congenital birth defect, affecting nearly 1% of all live births, and is the most common cause of infant death from birth defects. The aetiology of CHD in the majority of cases is unknown. Abnormal cardiac development appears to occur through a process that is heterogeneous and complex, with both environmental and genetic risk factors.^1^

The *NKX2-5* gene on chromosome 5q34 consists of two exons which encode a 324 amino acid protein. This homeobox transcription factor is expressed during early cardiac morphogenesis and serves as a master regulatory protein.^2^^–^4 Because of its critical role in cardiogenesis, *NKX2-5* has been a prime candidate in studies to identify the genetic basis of structural congenital heart defects. Mutations have been identified in patients with a variety of congenital heart malformations including septal defects, conotruncal abnormalities, cardiomyopathy, and hypoplastic left heart syndrome.^5^^–^7

Recent reports have suggested a novel genetic mechanism for CHD. Somatic mutations were identified in *NXK2-5* and its molecular partners, *TBX5* and *GATA4*, as well as the transcription factor *HEY2* in formalin fixed tissue taken from a collection of hearts with atrial septal defects (ASD),^8^ ventricular septal defects (VSD), and atrioventricular canal defects (AVCD).^9^^–^13 Somatic *NKX2-5* sequence variants were found in >95% of human hearts (66 of 68 hearts had a sequence variant) with septal defects and were detected by direct sequencing.^10^ ^13^ These sequence variants were primarily identified within malformed regions and not in unaffected regions taken from the same heart, indicating a somatic and mosaic nature for these variants. The investigators suggest that somatic sequence variants occur with high frequency and are aetiologic in cardiac malformations. Moreover, multiple *NKX2-5* sequence variants as well as multiple haplotypes were observed in a majority of these patients.^10^ ^13^ The same investigators also found somatic sequence variants in a number of other transcription factors such as *TBX5, GATA4,* and *HEY2,* often simultaneously within the same patient.^9^^–^13 The observation of two or more somatic sequence variants in the same patients and even within the same gene is expected to be an exceedingly rare event and would suggest a condition of extreme genetic instability, reminiscent of that observed in cancer.^13^

The somatic *NKX2-5* sequence variants were identified in DNA extracted from an archive of formalin fixed tissues that are at least 22 years old, the University of Leipzig (Germany) collection of malformed hearts.^9^ ^10^ ^13^ Because formalin can cause random base damage and affect polymerase chain reaction (PCR) fidelity,^14^ and degraded DNA or extremely low quantities of DNA from old frozen samples can increase risk for poor data quality,^15^ we were interested in replicating these experiments using fresh frozen cardiac tissue instead of formalin fixed tissue after decades of storage. We established a CHD tissue bank that houses blood and surgical discards from over 200 CHD patients. For this study, a cohort of 28 patients with both normal tissue samples and tissue samples within 1 mm of a septal defect were included. Genomic DNA (gDNA) was isolated from pathologic cardiac tissue and non-diseased cardiac tissue and blood collected from this cohort of CHD patients. gDNA from cardiac tissue samples was amplified by PCR and screened for *NKX2-5* mutations using direct sequencing and by a second more sensitive method, temperature gradient capillary electrophoresis (TGCE),^16^ to screen for any low frequency sequence variants that may not have been detected by direct sequencing.

## PATIENTS AND METHODS

### Patients

Institutional review board approval through the University of Louisville was granted both for the establishment of the CHD tissue bank and for this study. Written informed consent was obtained from all patients. Assent was obtained from paediatric participants when appropriate. Blood and surgical discards for DNA studies were obtained from the CHD tissue bank. All samples in this study were obtained from CHD patients undergoing cardiac surgery at Kosair Children’s Hospital (Louisville, Kentucky, USA) between March 2005 and March 2006. Tissue samples were obtained from surgical discards at the time of operation and from one heart explanted at the time of death (patient 18, table 1). Fourteen were female (50%) and 14 were male (50%). The mean age at time of surgery was 5.8 years (median 1.6 years, range 0–36.8 years).

**Table 1:** Summary of results (NKX2-5 exons 1 and 2)

Patient	Category	Diagnosis	Tissue samples	NKX 2.5 exon 1A			NKX 2.5 exon 1B			NKX 2.5 exon 2A			NKX 2.5 exon 2B		
				AA	Seq var	Zygos	AA	Seq var	Zygos	AA	Seq var	Zygos	AA	Seq var	Zygos
1	ASD	ASD (secundum)	Blood	Glu21Glu	A63G	het	None	None		None	None		None	None	
			ASD rim (1 mm proximity)	Glu21Glu	A63G	het	None	None		None	None		None	None	
2	ASD	ASD (secundum)	Blood	None	None		None	None		None	None		None	None	
			ASD rim (1 mm proximity)	None	None		None	None		None	None		None	None	
3	ASD	ASD (secundum)	Blood	None	None		None	None		None	None		None	None	
			ASD rim (1 mm proximity)	None	None		None	None		None	None		None	None	
4	ASD	ASD (secundum)	Blood	None	None		None	None		None	None		None	None	
			ASD superior rim (1 mm proximity)	None	None		None	None		None	None		None	None	
			ASD inferior rim (1 mm proximity)	None	None		None	None		None	None		None	None	
5	ASD	ASD (secundum)	Blood	Glu21Glu	A63G	het	None	None		None	None		None	None	
			ASD superior rim (1 mm proximity)	Glu21Glu	A63G	het	None	None		None	None		None	None	
			ASD inferior rim (1 mm proximity)	Glu21Glu	A63G	het	None	None		None	None		None	None	
6	ASD	ASD (secundum)	Blood	None	None		None	None		None	None		None	None	
			Superior ASD rim (1 mm proximity)	None	None		None	None		None	None		None	None	
			Inferior ASD rim (1 mm proximity)	None	None		None	None		None	None		None	None	
7	ASD	ASD (secundum)	Blood	Gln22Arg	A65G	het	None	None		None	None		None	None	
			ASD rim (1 mm proximity)	Gln22Arg	A65G	het	None	None		None	None		None	None	
8	ASD	ASD (secundum)	Blood	Glu21Glu	A63G	het	None	None		Gln181Gln	G543A	het	None	None	
			Superior ASD rim (1 mm proximity)	Glu21Glu	A63G	het	None	None		Gln181Gln	G543A	het	None	None	
			Inferior ASD rim (1 mm proximity)	Glu21Glu	A63G	het	None	None		Gln181Gln	G543A	het	None	None	
9	ASD	ASD (secundum)	Blood	None	None		None	None		None	None		None	None	
			ASD inferior rim (1 mm proximity)	None	None		None	None		None	None		None	None	
10	ASD	ASD (secundum)	Blood	Glu21Glu	A63G	het	None	None		None	None		None	None	
			ASD rim (1 mm proximity)	Glu21Glu	A63G	het	None	None		None	None		None	None	
11	ASD	ASD (secundum)	Blood	Glu21Glu	A63G	hom	None	None		None	None		None	None	
			ASD rim (1 mm proximity)	Glu21Glu	A63G	hom	None	None		None	None		None	None	
12	ASD	ASD (sinus venosus, PAPVR)	Blood	None	None		None	None		None	None		None	None	
			ASD superior rim (1 mm proximity)	None	None		None	None		None	None		None	None	
			ASD inferior rim (1 mm proximity)	None	None		None	None		None	None		None	None	
13	ASD	ASD (secundum, TAPVR)	Blood	Glu21Glu	A63G	het	None	None		None	None		Ala284Ala	C852G	het
			ASD inferior rim (1 mm proximity)	Glu21Glu	A63G	Het	None	None		None	None		Ala284Ala	C852G	het
14	VSD	VSD (perimembranous,	Blood	Glu21Glu	A63G	hom	None	None		None	None		None	None	
			VSD rim (1 mm proximity)	Glu21Glu	A63G	hom	None	None		None	None		None	None	
15	VSD	VSD (perimembranous)	Blood	None	None		None	None		None	None		None	None	
			VSD rim (1 mm proximity)	None	None		None	None		None	None		None	None	
16	VSD	VSD (perimembranous)	Blood	Glu21Glu	A63G	het	None	None		None	None		None	None	
			VSD rim (1 mm proximity)	Glu21Glu	A63G	het	None	None		None	None		None	None	
17	VSD	VSD (malalignment, TOF)	Blood	Glu21Glu	A63G	het	None	None		None	None		None	None	
			VSD rim (1 mm proximity)	Glu21Glu	A63G	Het	None	None		None	None		None	None	
18	VSD	VSD (perimembranous, D-TGA, HLH)	Right atrial appendage	Glu21Glu	A63G	hom	None	None		None	None		None	None	
			ASD (1 mm proximity)	Glu21Glu	A63G	hom	None	None		None	None		None	None	
			Anterior VSD rim (1 mm proximity)	Glu21Glu	A63G	hom	None	None		None	None		None	None	
			Posterior VSD rim (1 mm proximity)	Glu21Glu	A63G	hom	None	None		None	None		None	None	
			Anterior VSD rim (2–3 mm proximity)	Glu21Glu	A63G	hom	None	None		None	None		None	None	
			Posterior VSD rim (2–3 mm proximity)	Glu21Glu	A63G	hom	None	None		None	None		None	None	
19	AVCD	CAVC	Blood	None	None		None	None		None	None		None	None	
			ASD rim (1 mm proximity)	None	None		None	None		None	None		None	None	
20	AVCD	CAVC	Blood	Glu21Glu	A63G	het	None	None		None	None		None	None	
			ASD rim (1 mm proximity)	Glu21Glu	A63G	het	None	None		None	None		None	None	
21	AVCD	CAVC	Blood	Glu21Glu	A63G	hom	None	None		None	None		None	None	
			ASD rim (1 mm proximity)	Glu21Glu	A63G	hom	None	None		None	None		None	None	
22	AVCD	CAVC	Blood	None	None		None	None		None	None		None	None	
			VSD rim (1 mm proximity)	None	None		None	None		None	None		None	None	
23	AVCD	CAVC	Blood	Glu21Glu	A63G	het	None	None		None	None		None	None	
			ASD rim (1 mm proximity)	Glu21Glu	A63G	het	None	None		None	None		None	None	
			VSD rim (1 mm proximity)	Glu21Glu	A63G	het	None	None		None	None		None	None	
24	AVCD	CAVC	Blood	Glu21Glu	A63G	het	None	None		None	None		None	None	
			ASD rim (1 mm proximity)	Glu21Glu	A63G	het	None	None		None	None		None	None	
			VSD rim (1 mm proximity)	Glu21Glu	A63G	het	None	None		None	None		None	None	
25	AVCD	CAVC	Blood	Glu21Glu	A63G	het	None	None		None	None		None	None	
			ASD rim (1 mm proximity)	Glu21Glu	A63G	het	None	None		None	None		None	None	
			VSD rim (1 mm proximity)	Glu21Glu	A63G	het	None	None		None	None		None	None	
26	AVCD	CAVC	Blood	None	None		None	None		None	None		None	None	
			ASD rim (1 mm proximity)	None	None		None	None		None	None		None	None	
			VSD rim (1 mm proximity)	None	None		None	None		None	None		None	None	
27	AVCD	TAVC	Blood	None	None		None	None		None	None		None	None	
			ASD rim (1 mm proximity)	None	None		None	None		None	None		None	None	
28	AVCD	TAVC	Blood	Glu21Glu	A63G	het	None	None		None	None		None	None	
			ASD rim (1 mm proximity)	Glu21Glu	A63G	het	None	None		None	None		None	None	

AA, amino acid; ASD, atrial septal defect; AVCD, atrioventricular canal defect; CAVC, complete atrioventricular canal; D-TGA, D-transposition of the great vessels; het, heterozygous; HLH, hypoplastic left ventricle; homo, homozygous; PAPVR, partial anomalous pulmonary venous return; Seq var, sequence variation; TAPVR, total anomalous pulmonary venous return; TAVC, transitional atrioventricular canal; VSD, ventricular septal defect; Zygos, zygosity.

The study cohort included 28 unrelated patients with normal tissue samples and phenotypically abnormal tissue samples within 1 mm of the rim of ASD,^8^ VSD, and AVCD (table 1). This cohort was selected from the CHD tissue bank because they represented the same types of cardiac malformations included in the previous reports.^10^^–^14

### Sample size calculation and statistical analysis

Power calculations were performed for a test comparing two proportions where somatic *NKX2-5* sequence variants were observed in 66 out of 68 patients (n_1_ = 68 patients; p_1_ = 66/68) and n_2_ = 28 patients from this study. An observation of somatic variants in fewer than 22 patients would be considered significantly different from the previously published study reports (α = 0.05, β = 0.8).

### Sample collection and DNA isolation

Blood samples were acquired at the time of surgery. Tissue discards were snap frozen in liquid nitrogen and then stored in a freezer at −80°C. Genomic DNA was isolated from phenotypically normal and pathologic cardiac tissue and amplified using methods described previously.^13^ Genomic DNA was isolated from blood using QIAamp DNA Blood Midi Kit Protocol (Qiagen, Valencia, California, USA) and from 2–10 mg of frozen cardiac tissue samples using NucleoSpin Tissue Kit (Clontech Laboratories, Inc, Mountain View, California, USA).

### Amplification of NKX2-5 fragments

Previously published primer sequences were used for the amplification of the *NKX2-5* fragments 1A, 1B, 2A, and 2B.^5^ ^17^ The coding region of the *NKX2-5* gene, including exon/intron boundaries, was amplified from genomic DNA by PCR. PCR reactions consisted of 20–30 ng of genomic DNA, 3 μl of PCR buffer, 6 μl of 5X Q solution (Qiagen), 3 μl of dNTPs, 0.3 μl of Hotstar Taq (Qiagen), 1.5 μl (20 pmol/μl) of each primer pair, to a volume of 30 μl with distilled water. PCR reactions were undertaken on a Bio-Rad Thermal Cycler (Bio-Rad Laboratories Hercules, California, USA). Reactions started with 15 min at 95°C, followed by 40 cycles of 45 s at 95°C, 30 s at 60°C, and 45 s at 72°C, and finished with a 10 min extension period at 72°C.

### DNA sequencing

Double-stranded DNA fragments obtained from PCR reactions were purified with the MinElute PCR Purification Kit (Qiagen) using spin columns in a microcentrifuge. DNA samples were analysed by double-strand direct sequencing using *NKX2-5* specific primers. The sequencing reaction was performed using BigDye Terminator v3.1 Cycle Sequencing Kit (Applied Biosystems, Foster City, California, USA). Bidirectional sequencing was performed by an ABI 3730XL automated cycle sequencer (Applied Biosystems). Sequences were analysed using the software program, Sequencher v4.5 (Gene Codes Corp, Ann Arbor, Michigan, USA) as well as proof read by eye. The numbering of nucleotide changes was based on the reference sequence NM_002052 with +1 as the adenine nucleotide of the first codon ATG.

If a nucleotide sequence variation was found in DNA isolated from a pathologic cardiac tissue sample, then genomic DNA isolated from blood and/or phenotypically normal cardiac tissue samples obtained from the same patient was sequenced in an identical manner. Sequence variations found in genomic DNA isolated from pathologic cardiac tissue were then compared to sequencing results obtained from blood samples and normal heart tissues of the same patient.

### TGCE screening

To screen for sequence variants that might exist at frequencies lower than that expected to be detected by direct sequencing, a capillary electrophoresis based heteroduplex analysis platform (TGCE) was used (Spectrumedix System, Transgenomic Inc, Omaha, Nebraska). gDNA from pathologic cardiac samples and either blood and/or atrial appendage from all 28 patients was amplified by PCR and used to create heteroduplexes as previously described.^16^ TGCE analysis was performed on an automated 96 capillary array instrument (Spectrumedix System, Transgenomic Inc). DNA samples consisting of homoduplexes and heteroduplexes were separated by capillary electrophoresis, during which a thermal ramp from 50°C to 60°C was applied over 25 min; the injection conditions were 6 kV for 50 s using Reveal matrix (Spectrumedix System, Transgenomic Inc). SpectruMedix Check Mate Software was used for instrument control and data acquisition with a data acquisition time of 65 min. Data were analysed by use of the SpectruMedix Reveal Mutation Software as well as proof read by eye. The system generated the electropherograms for our reference sequence, the wild-type plus mutant (mixed) samples, and the mutant unmixed samples. Sequence variants were identified by automated detection of peak patterns where the presence of two peaks on an electropherogram indicated a nucleotide change.

## RESULTS

A total of 28 patients were selected for this study. They included: atrial septal defects (ASD = 13: 11 isolated secundum ASD, one sinus venosus ASD with partial anomalous pulmonary venous return, one secundum ASD with total anomalous pulmonary venous return); ventricular septal defects (VSD = 5: two isolated perimembranous VSD, one perimembranous VSD with right aortic arch and aberrant left subclavian artery, one perimembranous VSD with D-transposition of the great vessels, pulmonary atresia and hypoplastic left ventricle, one malalignment VSD with tetralogy of Fallot); and AV canal defects (AVCD = 10: eight with complete AV canal defects (CAVC), and two with transitional AV canal defects (TAVC)). Table 1 summarises the cohort of patients and the tissue samples obtained. Preoperative blood samples were available for 27 operative patients (blood was not available for patient 18). Right atrial appendage was available as a surgical discard because its removal was necessary before placing the patients on cardiopulmonary bypass; tissue samples from the right atrial appendage were available for 27 patients (atrial appendage was not available for patient 27). In total, 42 pathologic cardiac tissue samples were available for study.

### *NKX2-5* sequence variations

**Figure 1:**
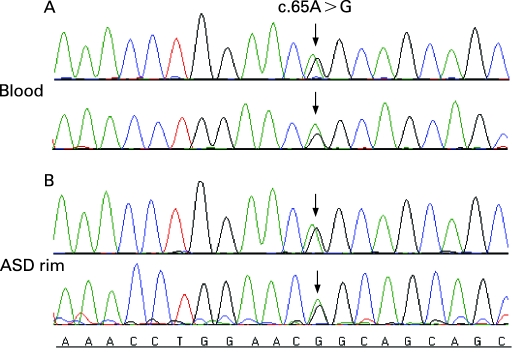
The *NKX2-5* heterozygous sequence variant c.65A>G (Q22R). The missense change was identified in the forward and reverse genomic DNA strands isolated from blood (A) and pathologic cardiac tissue (B) of the same patient. ASD, atrial septal defect.

Four single nucleotide changes were identified in 17 patients; one non-synonymous variation and three synonymous variations were found in the *NKX2-5* coding region (table 2). The non-synonymous sequence variant (65A>G) was heterozygous and was identified in one patient with an ASD (fig 1). This sequence variant results in a glutamine-to-arginine (Q22R) amino acid substitution, and is located on exon 1, just outside of the TN domain.

**Table 2:** Summary of *NKX2-5* mutations in patients with congenital heart defects

Nucleotide change	Predicted amino acid change	Location	ASD (+)	VSD (+)	AVCD (+)
Non-synonymous					
c.65A>G	p.Q22R	Exon 1	1		
Synonymous					
c.63A>G		Exon 1	6	4	6
c.543G>A		Exon 2	1		
c.852C>G		Exon 2	1		

ASD, atrial septal defects; AVCD, atrioventricular canal defect; VSD, ventricular septal defects.

Three separate synonymous sequence variations were found in 17 patients. The first is a common single nucleotide polymorphism (SNP), rs2277923 (63 A>G), and was identified in 17 patients. According to dbSNP, average heterozygosity of rs2277923 is 0.49±0.07. Heterozygosity of the CHD population in this study was 0.46 (13 of 28 patients). Seven of these patients had ASD (patients 1, 5, 7, 8, 10, 11, and 13), four had VSD (patients 14, 16, 17, and 18), and six had AVCD (patients 20, 21, 23, 24, 25, and 28). Thirteen of these patients were heterozygous, and four were homozygous for this variation. The second synonymous sequence variant, 543G>A, was found in one patient with a secundum ASD (patient 8). The patient was heterozygous for this nucleotide change. This patient was also heterozygous for the 63A>G SNP. The 543G>A variant has not been previously reported. The third sequence variant, 852 C>G, was found in one patient with a secundum ASD and total anomalous pulmonary venous return (patient 13). This patient was also heterozygous for this sequence variant and the 63 A>G SNP. We do not believe the 852C>G variant has been reported previously.

### TGCE results

TGCE results were consistent with the above sequencing results. No additional peaks representing potential additional mutations were detected in pathologic or normal cardiac tissues. The sensitivity of TGCE is better than direct sequencing; mutations present at 1 in 20 ratios are detected reliably.^16^

### Comparison with unaffected tissues

To determine if any of the sequence variations identified in pathologic tissue were germline or somatic, blood samples and phenotypically normal heart tissues were also screened from the same patient. We analysed 50 sequence variations discovered in both normal and pathologic tissues in seven patients with ASD (patients 1, 5, 7, 8, 10, 11, and 13), four patients with VSD (patients 14, 16, 17, and 18), and six patients with AVCD (patients 20, 21, 23, 24, 25, and 28). Every nucleotide sequence variant found in the pathologic tissue samples was also found in the normal cardiac tissue or blood samples taken from the same patient—indicating that these were germline variants. We found no evidence of *NXK2-5* somatic sequence variants in any of our samples.

## DISCUSSION

There is convincing evidence that germline *NKX2-5* mutations are aetiologic in CHD.^5^^–^7 The aim of our current study was to replicate the previous finding of somatically derived sequence variants in patients with septal defects.^10^ ^13^ In these published reports, somatic *NKX2-5* sequence variants were primarily identified within malformed regions and were not seen in unaffected regions of the heart; taken together, these findings suggest that somatic mutations occur with high frequency and are also aetiologic in CHD.^10^ ^13^

These remarkable reports come from DNA isolated from archival tissues after more than 22 years of formalin storage. Formalin is known to create PCR artefacts that may appear as mutations. The purpose of our study was to replicate these controversial findings using fresh frozen cardiac tissue instead of >22-year-old formalin fixed tissue. We sequenced genomic DNA isolated from pathologic cardiac tissues collected as surgical discards from patients with CHD and searched for *NKX2-5* mutations. We compared our finding with sequencing results using DNA from concurrently obtained non-diseased cardiac tissue and blood samples. We identified three novel germline *NKX2-5* sequence variants in our cohort of 28 unrelated CHD patients. One ASD patient was found to have a heterozygous sequence variant located just outside of the TN domain and results in an amino acid substitution. Two unique silent sequence variants were identified in two patients who also carried a common SNP. The common SNP was identified in 16 patients. No evidence of somatic *NKX2-5* sequence variants was found in any of the samples described herein.

In our study, an average of 1.3 μg of genomic DNA (gDNA) was isolated from 5 mg of tissue obtained as surgical discards that were immediately frozen. In comparison, yields from the Leipzig collection were reportedly 0.5–1 μg of genomic DNA from 25 mg DNA obtained from archival tissue. Therefore, the recovery of gDNA from our fresh frozen samples was more than five times that of the formalin fixed samples. The relatively poor gDNA yield from the Leipzig collection likely reflects the poor quality of DNA taken from samples that had been fixed in formalin between 22–50 years.

### Mutational spectra comparisons

The Human Gene Mutation Database^18^ is an up-to-date and comprehensive collection of published germline mutations in nuclear genes underlying or associated with human inherited disease.^19^ The database is maintained by the Institute of Medical Genetics in Cardiff and is available online at www.hgmd.org. It contains statistics on 34 066 germline single nucleotide substitutions. Guanine-to-adenine (G>A) mutations dominate the database. The majority (44%) of single nucleotide substitutions have been reported to be G>A variations. The second most prevalent sequence variation is an adenine-to-guanine (A>G) substitution (17%).

The International Agency for Research on Cancer (IARC) TP53 Mutation Database is a compilation of all *P53* gene mutations identified in human cancers and cell lines that have been reported in the peer review literature since 1989.^20^ It is available online at www.iarc.fr/P53/. The R10 release of the Somatic Mutation Dataset contains 21 587 mutations associated with human cancers. Of these, there are 18 643 single nucleotide substitutions. Again, G>A nucleotide substitutions comprised the majority (51%) of the reported sequence variations. Only 13% of the *P53* somatic variations were A>G substitutions.

To date, there are 60 germline *NKX2-5* single nucleotide substitutions that have been published in the peer review literature.^6^ The vast majority (61%) of these are also G>A substitutions. A>G substitutions have been reported in only 2% of these sequence variations.

Six hundred and five somatic sequence variants were reported in the coding and flanking intron regions of *NKX2-5*.^10^ ^13^ When we analysed the spectrum of these sequence variants, found after amplification from DNA extracted from formalin fixed tissue, we were surprised to discover that A>G substitutions dominated these studies (58% of these sequence variants were A>G substitutions) and only 31% were G>A substitutions.

Similarly, the same group found somatic sequence variants in the *TBX5* gene from the same archival collection of hearts. *TBX5* encodes a transcription factor that interacts with *NKX2-5* and is important in vertebrate cardiac development.^21^ ^22^ One hundred and thirty-five somatic sequence variations have been reported by the same authors in the coding and flanking intron regions of *TBX5*.^9^ Of the single nucleotide substitutions reported, 87% were adenine-to-guanine substitutions. Only 11% were guanine-to-adenine substitutions.

MITOMAP is a comprehensive database of human mitochondrial DNA (mtDNA) variation and its relationship with human evolution and disease.^23^ It is available online at www.MITOMAP.org. MITOMAP maintains a compendium of all known pathogenic mtDNA mutations. The MITOMAP Somatic Mutation database (updated 6 April 2006) contains 135 single base substitution mutations. The MITOMAP Coding and Control Region Point Mutation database (updated 18 May 2006) contains 277 single base substitution mutations. Although dominant mutational pathways in the mitochondria may be quite different from mutational pathways in nuclear genes, again, G>A nucleotide substitutions comprised the majority of the reported sequence variations in both the germline and in somatic mtDNA mutations (46% and 44%, respectively). A>G substitutions were found in 39% of the reported germline mutations and 43% of the somatic mutations.

**Key points**

- Somatic mutations in NKX 2.5 were not found in any fresh frozen diseased tissue sample from 28 patients with cardiac septal defects.
- Somatic mutations in NKX 2.5 are not a major aetiological pathway in cardiac septal defect formation.
- Low yield or poor template DNA quality may account for the apparent observations of somatic mutations in previously published reports.
- Mutational spectra are an important consideration in somatic mutation analysis.

**Figure 2:**
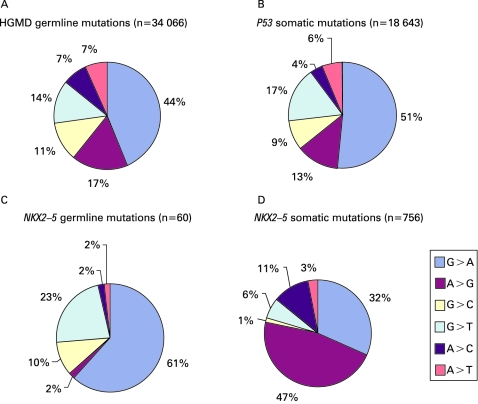
Mutational spectra comparisons. Guanine-to-adenine nucleotide substitutions are the most frequently reported sequence variation in the germline Human Gene Mutation Database (HGMD) (A), the International Agency for Research on Cancer (IARC) *TP53* Somatic Mutation Database (B), and previously published *NKX2-5* germline mutations (C). Adenine-to-guanine substitutions are the most common sequence variant reported in previously published *NKX2-5* somatic mutations (D).^10^ ^13^

**Figure 3:**
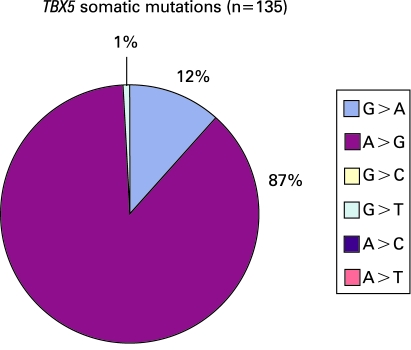
Mutational spectra of somatic TBX5 mutations. Adenine-to-guanine nucleotide substitutions are the most common sequence variant reported in previously published *TBX5* somatic mutations.^9^

**Figure 4:**
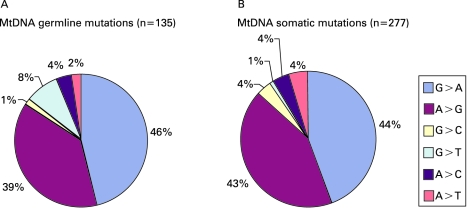
Mutational spectra of mitochondrial mutations. Guanine-to-adenine nucleotide substitutions are the most frequently reported sequence variation in germline mitochondrial DNA (A) and somatic mitochondrial DNA (B).

These analyses, summarised in figs 2–4, demonstrate that the somatic mutational spectra from the Reamon–Buettner studies are significantly different from germline *NKX2-5* mutations as well as significantly different from inherited disease associated mutations, tumour derived somatic mutations, and mitochondrial mutations. While these differences may reflect true differences between the patients at the mutational pathway level (germline versus somatic versus mitochondrial mutations), geographic and/or patient level (Germany and/or CHD patients versus an international collections of cancer databases), or the temporal level (over 22 years ago versus the current era), it is also possible that the previously reported somatic variants reflect postmortem artefacts of fixation or low quality DNA template. Recent studies also show that lower starting yield of genomic DNA from archival samples can result in higher misincorporation rates during PCR in a sequence dependent manner.^24^

Much work needs to be done to define the genetic basis of CHD. The complex relationship between environmental stresses, genetic mutations and elaborate molecular mechanisms which lead to structural heart defects remain to be elucidated. We were unable to replicate the previously published findings of somatically derived sequence variants when fresh frozen cardiac tissue was used instead of >22-year-old formalin fixed tissues. We identified novel germline *NKX2-5* sequence variants in our CHD cohort, but we found no evidence of somatic variants using direct sequencing. More sensitive mutation detection approaches may be necessary to see low frequency somatic variants. However, the somatic mutation data from the previously published studies were obtained by conventional sequencing. We have shown that our recovery of genomic DNA from fresh frozen samples was five times greater than the gDNA recovery from that study which used formalin fixed samples. Furthermore, we have shown that the somatic *NKX2-5* mutational spectrum from the previous study is quite different from other germline and somatic mutation databases. We hope this work both elevates the discussion regarding somatic mutations and the aetiology of CHD and challenges the interpretation of genetic data obtained from formalin fixed archival tissues.
